# 3D printing of plasmonic nanofocusing tip enabling high resolution, high throughput and high contrast optical near-field imaging

**DOI:** 10.1038/s41377-023-01272-6

**Published:** 2023-09-06

**Authors:** Li Long, Qiurong Deng, Rongtao Huang, Jiafang Li, Zhi-Yuan Li

**Affiliations:** 1 School of Physics and Optoelectronics, South China University of Technology, Guangzhou, 510641 China; 2 School of Physics, Beijing Institute of Technology, Beijing, 100081 China; 3 State Key Laboratory of Luminescent Materials and Devices, South China University of Technology, Guangzhou, 510640 China

**Keywords:** Optical techniques, Laser-produced plasmas

## Abstract

Scanning near-field optical microscopy (SNOM) offers a means to reach a fine spatial resolution down to ~ 10 nm, but unfortunately suffers from low transmission efficiency of optical signal. Here we present design and 3D printing of a fiber-bound polymer-core/gold-shell spiral-grating conical tip that allows for coupling the inner incident optical signal to the outer surface plasmon polariton with high efficiency, which then adiabatically transport, squeeze, and interfere constructively at the tip apex to form a plasmonic superfocusing spot with tiny size and high brightness. Numerical simulations and optical measurements show that this specially designed and fabricated tip has 10% transmission efficiency, ~ 5 nm spatial resolution, 20 dB signal-to-noise ratio, and 7000 pixels per second fast scanning speed. This high-resolution, high throughput, and high contrast SNOM would open up a new frontier of high spatial-temporal resolution detecting, imaging, and monitoring of single-molecule physical, chemical, and biological systems, and deepen our understanding of their basic science in the single-molecule level.

## Abstract (web summary)

3D printing of a fiber-bound polymer-core/gold-shell spiral-grating conical tip that has 10% transmission efficiency, ~ 5 nm spatial resolution of optical near-field imaging, 20 dB signal-to-noise ratio, and fast scanning speed.

## Graphical abstract

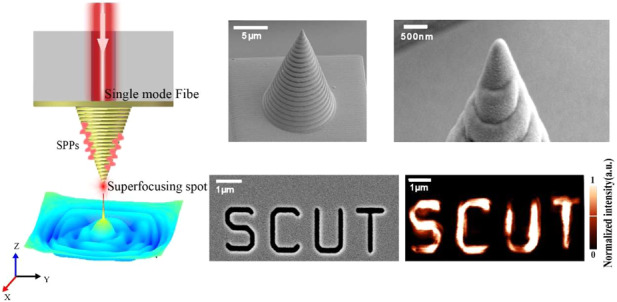

## Introduction

Optical microscopy offers a bridge connecting macroscopic and microscopic world through imaging, spectroscopy, and other means in space-time domain. But it also determines the spatial resolution of optical imaging to be micrometer scale at best, thanks to the diffraction effect of light wave, and this becomes the biggest disadvantage of conventional optical microscopy. In recent decades there has been increasing demand to lift this limitation and push the resolution down to nanometer scale, thus effectively upgrading optical microscopy into optical nanoscopy. Meanwhile, it is also highly desired that the advancement in the imaging resolution of nanoscopy is not in the price of severe degradation in many other advantages of conventional optical microscopy. History tells us that this is never an easy task. Among different means of super-resolution optical imaging schemes and technologies, scanning near-field optical microscopy (SNOM) is a purely optical technique that can effectively lift the diffraction limit of resolution^1–4^. In early 1980s, the great success of scanning tunneling microscopy (STM) and atomic force microscopy (AFM) stimulated the invention of SNOM^5–9^, and since then a wide variety of SNOM instruments have been developed, advanced, and placed into applications for basic sciences^10,11^.

**Fig. 1:**
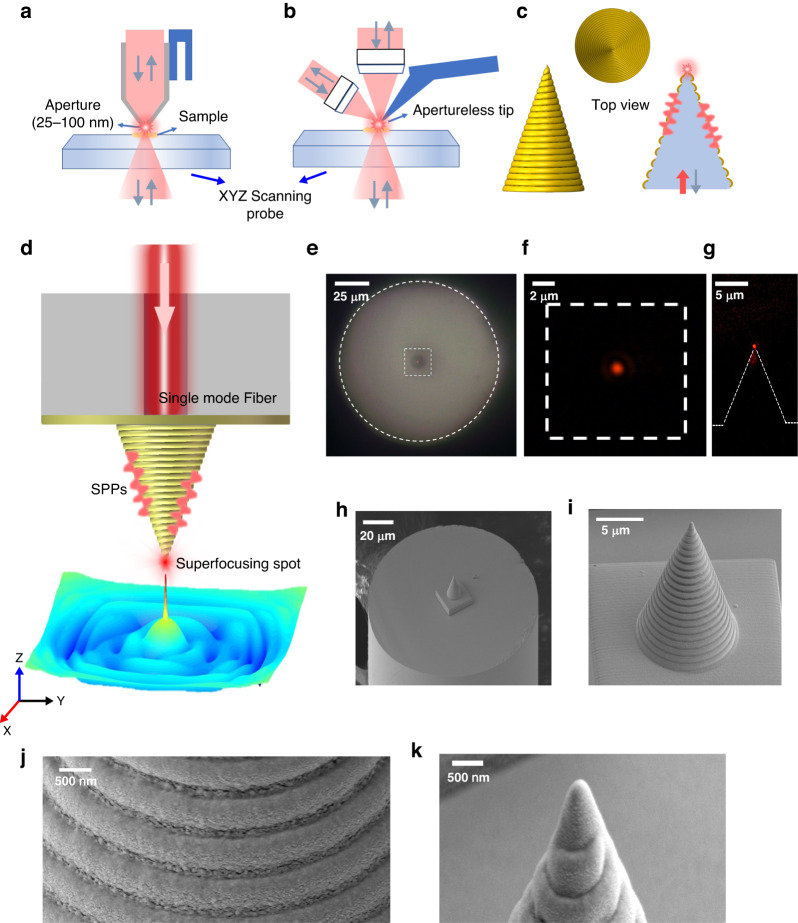
Principle for design and fabrication of plasmonic nanofucsing tip for SNOM near-field imaging. **a** Principle of operation for a classical a-SNOM. **b** Principle of operation for a classic s-SNOM. **c** Principle of operation for the current gold spiral-grating conical tip. **d** Schematic of plasmon nanofocusing conical tip directly built on the facet of optical fiber to serve as a high-performance SNOM tip under internal illumination. **e** Top-view optical microscope image of the fabricated SNOM tip. The outer dashed circle means the optical fiber edge, and the inner dashed square denotes the location of the center conical tip. **f**, **g** Top view and side view respectively of the optical image against a nanofocusing light spot at 785 nm emitting from the tip apex, as observed by a dark-field microscope objective. **h**–**k** Scanning electron microscopy (SEM) image of the fabricated spiral-grating conical tip in different scale ranges of view

The classical SNOM techniques can be classified into the “aperture SNOM” (a-SNOM)^3–8^ and “aptertureless or scattering SNOM” (s-SNOM)^9,12–19^ categories. As schematically illustrated in Fig. 1a, a-SNOM can have high resolution and high contrast by reducing the aperture size, but suffers low throughput, while the s-SNOM, as schematically illustrated in Fig. 1b, can have high resolution by reducing the tip apex size, but suffers both low throughput and low signal contrast. Many schemes have been developed and adopted to relieve the difficulty of very low transmission efficiency intrinsic with classical a-SNOM and s-SNOM instruments. In regard to metal-coated tapered fiber tip in a-SNOM, Xu et al. adopted complex-shape aperture such as H-shaped and bowtie-shaped aperture other than regular circular aperture and the transmission efficiency could increase by one order of magnitude^20^. Zhang et al. integrated a 100 nm nanoaperture surrounded by a circular through grating that resembles a plasmonic lens on the metal coating of a conic SNOM tip^21^. With a balance of luminous flux and aperture size, these deliberately designed probes still have very low optical throughput, resulting in weak signal light in use. Besides, the resolution is limited due to aperture size, at about 50-100 nm.

Two major problems exist for apertureless s-SNOM, one is the weak transmission efficiency of signal light for use, the other is the strong background signal and thus bad signal-to-noise ratio (SNR) level. Ropers et al. introduced a one-dimensional (1D) grating on the shaft of a sharp conical metal taper with a tip radius of few tens of nanometers. This grating can help to transform the incident far-field laser beam into resonant generation of SPPs traveling over more than 10 µm to the tip apex and converging to an intense radiative local light spot with size of few tens of nanometers. The efficiency is significantly enhanced to the level of 0.1%-1%, and the background signal is greatly suppressed and SNR level is increased a lot, opening better opportunities in nanospectroscopy^22,23^. An alternative scheme is to create 3D metal tip with patterned metallic pyramids obtained via silicon template stripping. Gratings on the faces of these pyramids can convert broad area of incident linearly polarized light into plasmons that propagate toward and converge at a ~10 nm apex with a focus spot with size of few tens of nanometers^24^. This broad-area illumination light brings about a relatively high efficiency of focusing but in the price of degraded SNR^25,26^. Li et al. demonstrated an efficient scheme to remove background signal by designing and building a spiral-grating conical Au tip, where the signal light is internally input to illuminate the tip and efficiently excite SPPs at the outer surface via the corrugation metal grating to propagate down to the tip apex to form a 3D nanofocusing plasmonic spot with few tens of nanometers in size^27^.

More recently, Jiang et al. designed and demonstrated a plasmonic lens tip (p-tip) on a commercial SiO_2_ AFM probe without a nanoaperture at the tip apex^28^. Subwavelength circular gratings composed of six annular slits on the 120 nm thick outer coating Au film were fabricated to couple the internal radial polarized illumination to SPPs that led to an ultrastrong, superfocused spot via adiabatic nanofocusing. It was claimed that this design could render an optical resolution of 10 nm, a throughput of 3.28%, which is approximately four orders higher than a commercial a-SNOM tip (a-tip), and an outstanding signal noise ratio of up to 18.2 (nearly free of background). Yet, this p-tip is specially designed to match the radial-polarization vectorial laser beam, but not for more general polarization illumination states, somewhat leading to limitation upon practical usage.

The above lessons learned and accumulated in the past two decades have taught us quite a lot about the strategy towards reaching a balanced high performance of SNOM in terms of spatial resolution, transmission efficiency, and background signal level. And here we eventually accomplishing 3H (high resolution, high throughput, and high contrast or SNR) SNOM tip. We present a successful design and realization of an efficient solid-core spiral-grating conical gold tip, as schematically illustrated in Fig. 1c, which can accomplish excellent overall performance with high resolution (~5 nm), high throughput (~10%), and high contrast (~20 dB SNR) when introduced into conventional SNOM machine. This tip has several merits. First, the signal light comes from the optical fiber and internally illuminates the inner side of the spiral-grating tip. Second, the gold spiral grating conformally coated on the solid polymer core template can offer flexible momentum matching condition to excite SPPs. Third, the lack of geometric symmetry guarantees that the excited SPPs from various azimuthal channels at the surface of the spiral-grating conical tip can constructively interfere at the tip apex to form a highly localized nanofocusing spot with high brightness. This novel SNOM tip can find potential important applications in high-resolution optical imaging, high-sensitivity optical detection, Raman spectroscopy, photoluminescence, molecular fluorescence, and other nanoscale spectroscopy studies.

## Results

### Design of spiral-grating SNOM tip

**Fig. 2:**
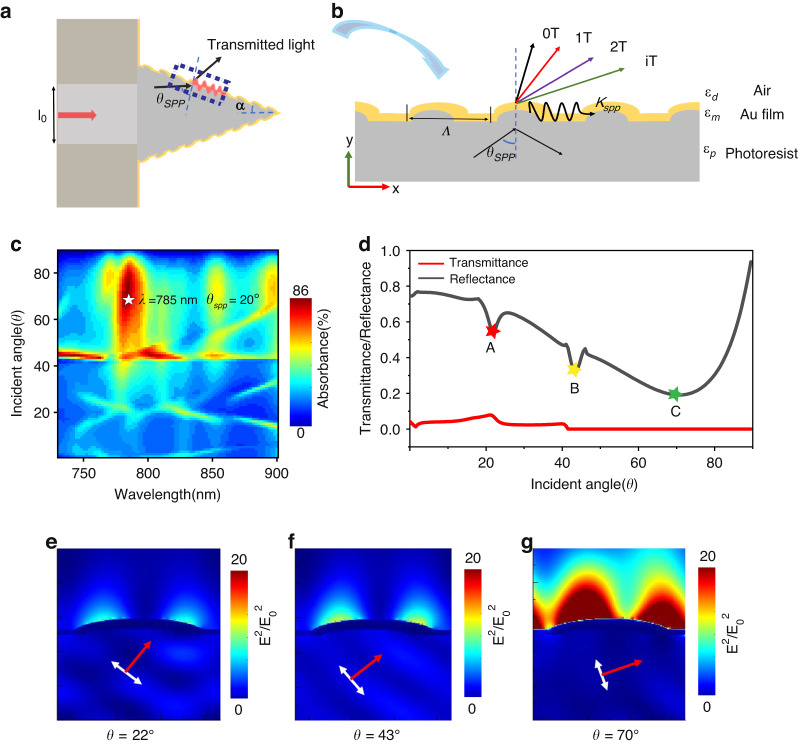
1D design model of high-efficiency plasmon excitation to assist design of 3D spiral-grating conical tip. **a** Schematic diagram of the spiral-grating helical probe at the yz section. **b** One-dimensional enlarged view of the grating structure corresponding to the dotted box in (**a**). **c** Calculated absorbance of the grating structure versus different incident angles and incident wavelengths. Color-bar represents the strength of absorbance. **d** Calculated transmittance and reflectance at different incident angles for the incident light wavelength of 785 nm. **e**–**g** The simulation results of the electromagnetic field intensity distribution of the 1D grating structure corresponding to the three points A (*θ* = 22^*°*^, *λ* = 785 nm), B (*θ* = 43^°^*, λ* = 785 nm), and C (*θ* = 70^°^*, λ* = 785 nm) in (**d**). The colorbar represents the electric field intensity

With reliable and precise 3D printing nanofabrication technique at hands, first step is to explore and find appropriate geometric parameters, the half conical angle *α* and height *h* of the tip, the pitch *Λ*, depth *d* and corrugation shape of spiral-grating, the thickness *t* of gold thin film, for spiral-grating conical tip with balanced high performance of resolution, throughput, and contrast. We are confronted with a six-dimensional parameter space. Surely we have a huge choice of freedom, but also a huge burden. Therefore, we considering this dominant process of grating-assisted SPPs excitation, simplify the complex 3D helix tip structure into a 1D grating SPPs coupling device model, as illustrated in Fig. 2b. We scan the wavelength and angle of the incident light and calculate the reflection, transmission, and absorption of incident light against the metal grating by adopting the rigorous coupled-wave theory^29^. For more details of calculation, see Supporting Information, Section S2. The calculation results are shown in Fig. 2c. The reflectance is the lowest when the incident light wavelength is 785 nm and the incident angle is 70°. Thus we judge *θ*_*SPP*_ = 70^*o*^ and *λ*_*SPP*_ = 785nm, and at this condition, more energy is coupled to the outer surface of the metal grating to form SPP evanescent waves. For more design of spiral-grating SNOM tip see Supporting Information, Sec. S3.

In order to verify the correctness of the rigorous coupled-wave theory calculation, we use the FDTD method to calculate the 1D electromagnetic field displayed in Fig. 2e–g, respectively, see Supporting Information, Section S4. And 3D-FDTD electromagnetic field are displayed, which calculation results are presented and discussed in Methods, Section S5 and Figs. S2–4. We believe that the high throughput of this SNOM tip can be largely attributed to the usage of dual SPP excitation methods: the prism coupling and grating coupling, in the single SNOM tip. The collective action of these two powerful SPP excitation methods have significantly improved the transmission efficiency of optical energy from inner polymer core to the outside air-gold SPP mode.

### Experimental characterization of SNOM tip throughput and contrast

After theoretically and numerically determining the optimum conditions for 3D spiral-grating conical tip, we proceed to build and characterize such a tip. The geometry of the 3D printing conical tip has been illustrated in Fig. 1h–k, according to the detailed design presented in Methods, Section S3 and Fig. S1. The size of the solid-core spiral-grating conical tip has a bottom diameter 10.2 μm, a cone height 14 μm and half conical angle 20^o^. The nanostructured tip is built via two-photon polymerization direct-laser writing 3D printing technique^30,31^, see Supporting Information, Section S1. The preliminary optical characterization of the tip has already been discussed in Fig. 1e–g, which show that there only exists an output channel of light signal right at the center of the tip, but the precise performance index of resolution, throughput, and contrast still needs a thorough experimental examination.

The signal throughput for the solid-core spiral-grating conical tip is displayed in Table 1 at different input signal powers, which is calibrated as equal to the output power of optical fiber of flat-cleaved facet. For more detail of SNOM tip throughput and contrast see Supporting Information, Section S6. The efficiency (namely, the throughput) of the tip fluctuates between 9.8% and 8.8% at different input power levels (different laser powers can cause thermal effects on the photoresist, resulting in changes in structural configuration and slightly reduced throughput), assuming an average throughput of 9.3%, which is nearly three times the throughput reported in Ref. ^28^. Table 1 also shows that the throughput of the hollow-core tip is only 1.4%, already not a bad number, but far smaller than the solid-core tip, indicating the importance of design and optimization in both geometric configurations and material composites. The major reason is the current configuration of hollow-core tip does not support good momentum matching condition for efficient SPPs coupling.

**Table 1:** Simulation and measurement of SNOM tip throughput

Tip	Input power (nW)	Output power (nW)	Throughput
Hollow-core tip	705	10	1.4%
Solid-core tip (Experiment)	85	8.33	9.8%
	132.5	12.77	9.6%
	157.3	14.2	9.0%
	187	16.5	8.8%
Solid-core tip (Simulation)			19.7%

### Experimental characterization of SNOM tip resolution

**Fig. 3:**
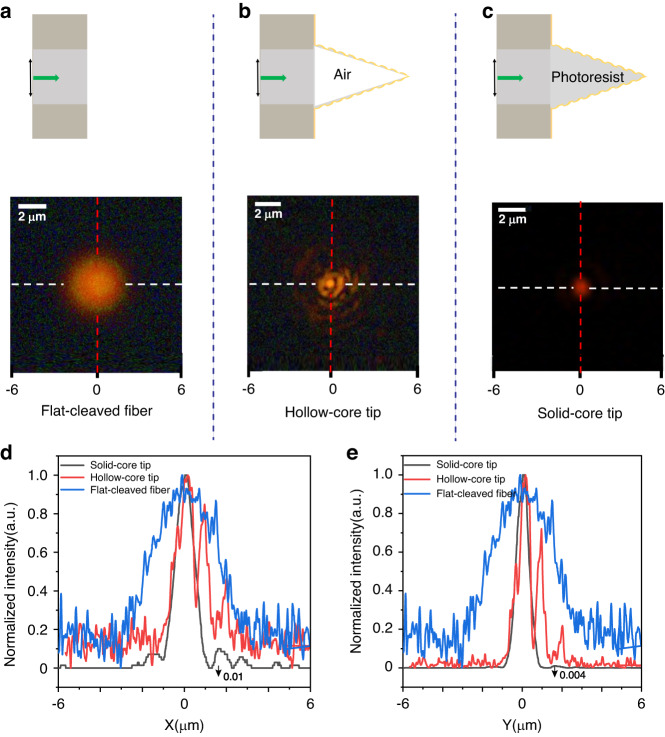
Schematic diagram and the far field modal field profile of three tip samples observed by CCD camera. **a** The flat-cleaved fiber, **b** the hollow-core tip, and **c** the solid-core tip. **d**, **e** The 1D curve of mode field profile along the two cross dashed lines, with white lines along the X and red lines along the Y direction, for the three samples

**Fig. 4:**
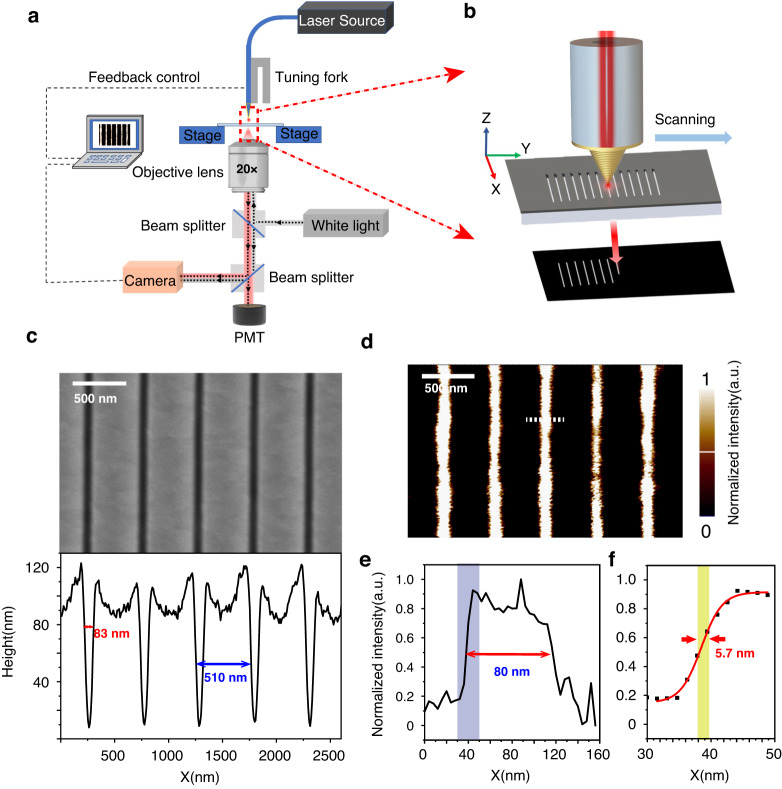
Experimental characterization of home-made SNOM tip resolution by performing near-field imaging upon a standard chrome lamellar-slits grating. **a** The experimental layout for SNOM imaging and (**b**) zoomed-in region of the SNOM probe and the sample. **c** The SEM image of the standard chrome grating sample sculptured into a 60 nm chromium thin film (upper panel), and the 1D enlarged image plotted along the dotted line marked in the SEM image, which shows that the period is 510 nm and the slit width is 83 nm. **d** 2D SNOM near-field imaging picture against the standard chrome grating sample. **e** 1D SNOM near-field imaging curve scanned along the white dashed line marked in (**d**). **f** 1D curve for the magnified area of the blue shaded part in (**e**), which shows that the resolution is ~5.7 nm

Because of the diffraction limit to imaging resolution, the far-field optical microscopic images presented in Figs. 1f, g and 3c are not sufficient to determine the accurate value of the focusing spot size of the solid-core tip. This size must be measured by adopting the powerful technique of SNOM near-field imaging, because according to the calculation presented in Section S7 and Fig. S5, the SNOM resolution is approximately equal to the size of nanofocusing spot used to illuminate a sample in the near field region as 10 nm. Keeping in mind this simple relationship, we carry out systematic experiments. We replace the 100 nm-resolution AFM-like tip equipped in a commercial SNOM instrument (NTEGRA Solaris SNOM, NT-MDT) by our home-made spiral-grating conical tip. Figure 4a–b shows a schematic diagram of the experimental setup and a magnified layout of the sample and SNOM tip used for optical near-field imaging. The SNOM operates in the illumination mode. For more details of near-field imaging, see Methods Section S8 and Fig. S6.

The spatial resolution in microscopy is often assessed by measuring the width of a typical lamellar line groove spanning a sharp boundary between two different materials. In our work, we use a grating composed of periodic slits (line grooves) perforated through a chrome film of 60 nm in thickness as the standard sample for SNOM imaging and evaluation of its spatial resolution. The slits are sculptured using focused ion beam (FIB) lithography. The SEM image of the standard sample is shown in Fig. 4c. The grating period is 510 nm, and the slit width is 83 nm, as found from the 1D curve of SEM picture. Chrome is a bad metal strongly absorptive of light, has bad reflection and nearly zero transmission, and usually shows grey to dark color in eye view. There is almost no multiple-scattering between the chrome sample and our gold conical SNOM tip, and this avoids undesirable complication in the near-field image interpretation that is commonly encountered in most SNOM imaging experiments. Ideally this standard sample has an infinitely sharp edge of air and chrome in geometry, and should also show a sharp edge of bright and dark region with zero cross-over distance *w* in the SNOM imaging with ideally infinitely fine resolution. But when the SNOM has finite resolution, the sharp edge would has a smooth cross-over distance from dark to bright region in the image. In more mathematical terms, the line groove profile can be thought of as the so-called edge response function (ERF), and the characteristic width *w* of an ERF is known as the line spread function (LSF). The LSF represents an image of a linear object, usually a Gaussian function centered on the material boundary. The width of the LSF determines the resolution according to some specific standard such as Rayleigh criterion popularly used to evaluate the resolution of conventional optical microscope, which is just about half wavelength. Careful theoretical calculations presented in Methods Sec. S7 and Fig. S5 indicate that the resolution of SNOM is nearly equal to the size of the SNOM tip nanofocusing spot used to illuminate the standard chrome grating sample. Based on these theoretical knowledges, we have the capability to determine the nanofocusing spot size as well as the SNOM imaging resolution.

The standard chrome grating sample is observed by our home-modified SNOM instrument armed with the gold spiral-grating conical tip operating in the illumination mode. The near-field optical imaging is made at a scanning speed of 15 μm s-1 and a step size of 2 nm, corresponding to a scanning pixel speed of 7000 pixel s-1 and 0.13 ms per pixel. This is a very fast near-field image acquiring speed and it can be only attributed to the ~10% high throughput of the SNOM tip. The 2D pictures of near-field scanning image obtained for this standard chrome grating sample is shown in Fig. 4d. The bright region for each perforated slit domain and dark region for other intact chrome thin film domains is distinct. According to the edge curve distribution of Fig. S5b in the supporting material, we use the Boltzmann distribution to fit the experimental data. The 1D scanning curve of near field picture across a slit as displayed in Fig. 4e-f shows a near-field imaging resolution of about 5.7 nm for this SNOM tip, which clearly shows that this tip can push the SNOM resolution well below 10 nm.

**Fig. 5:**
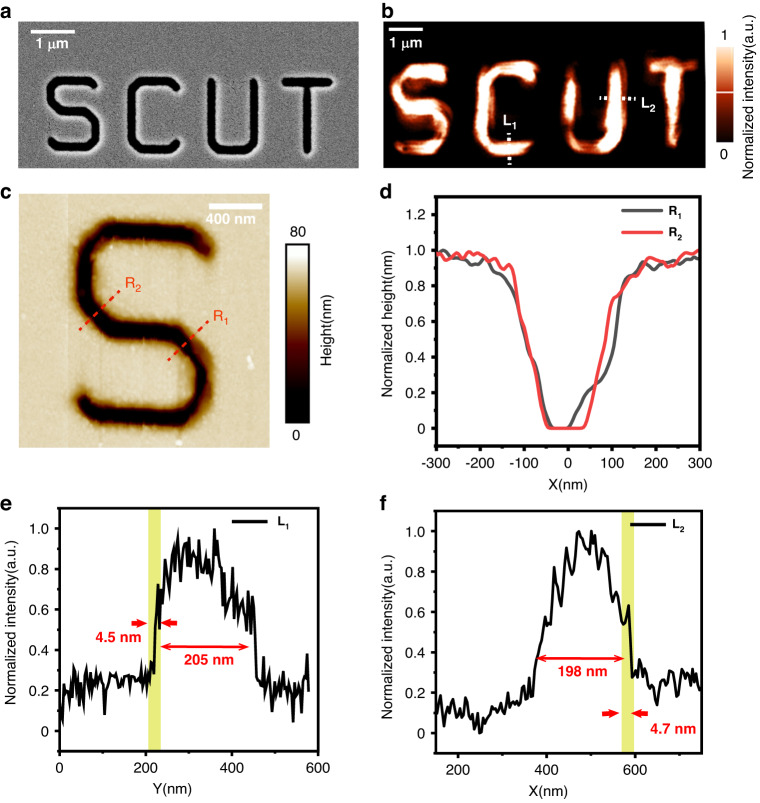
Experimental characterization of home-made SNOM tip resolution by performing near-field imaging upon 2D letters of lamellar slits perforated in chrome thin film. **a** SEM image of the “SCUT” logo pattern sample sculptured and perforated into 80 nm thick chromium film. **b** The SNOM scan image for the “SCUT” logo sample. **c** AFM height image engraved with the letter “S”. **d** AFM image for the 1D profile taken along the white dotted lines R_1_ and R_2_ as marked in (**c**). **e**, **f** 1D near-field image curves scanned along white dotted lines L_1_ and L_2_ as marked in (**b**)

To further demonstrate the excellent performance of our home-made SNOM tip, including its independence upon the incident light polarization, we change the standard sample to a logo pattern composed of four letters as “SCUT” perforated into a 80 nm thick chrome thin film, again by using the FIB lithography. Each letter consists of line grooves (slits) orienting in different directions, including the orthogonal X and Y direction. The SEM image is shown in Fig. 5a, where the slit width is set to be 198 nm. A typical 2D SNOM imaging picture recorded at the same scanning speed of 15 μm s-1 and scanning step of 2 nm for this complicated sample is displayed in Fig. 5b. Since in principle SEM can only reflect the surface morphology of the sample, it cannot disclose the detailed structure inside the sample slit. To determine this subtle local 3D geometric configuration, we image the slit with a standard AFM instrument. The 2D AFM picture for the letter “S” is shown in Fig. 5c, and the 1D curve representing two white dotted lines R1 and R2 across the letter are displayed in Fig. 5d. There appear apparent unevenness at the edges of the FIB sculptured lines, indicating that the slits do not have ideally sharp edges in geometry, but rather involve smooth cross-over regions. This geometry apparently will cause some pattern unevenness in the SNOM imaging picture that has been illustrated in Fig. 5b. The existence of the cross-over region within a slit makes the SNOM image of this slit, a bright line, narrower than the ideal slit with sharp edge and no cross-over region, in good agreement with the observed SNOM picture. This confirms once again that this home-made SNOM tip indeed has a very fine resolution so that it can reveal clearly such kind of 3D geometric unevenness within the inner space of a slit.

To accurately examine the resolution of the home-made SNOM tip against the complex pattern, we select two cross sections within the letters as marked in Fig. 5b, one is L_1_ along the vertical Y direction, and the other is L_2_ along the horizontal X direction. The corresponding 1D curves plotting the near-field imaging data are displayed in Fig. 5e, f. The results show that the spatial resolution in the 2D scan for the complex 2D logo pattern can reach a very high value up to 4.5 nm in Y direction and 4.7 nm in X direction. No wonder this tip can reveal the geometric unevenness within practical slits sculptured within the chrome thin film in a level matching up the powerful classical AFM instrument. When looking back at the near-field imaging picture displayed in Fig. 4d for 1D grating slits, the unevenness within the picture, i.e., some parts of slit wider and some parts of slit narrower, now can be attributed to the geometric unevenness in various regions of slits across the practical grating sample perforated in the chrome thin film. These data suggest the FIB nanofabrication technology should be improved to provide better standard samples for more reliable SNOM experiments.

## Discussion

In summary, we have presented the design and 3D printing nanofabrication of a fiber-bound polymer-core/gold-shell spiral-grating conical tip to serve as SNOM near-field imaging probe, in a wish to achieve simultaneously high resolution, high throughput, and high contrast that are highly desirable but very difficult to accomplish in previous schemes of a-SNOM and s-SNOM. Our design of SNOM tip is to introduce a gold spiral grating at the surface of dielectric conical tip and help to offer flexible momentum matching condition for coupling the inner optical signal coming from optical fiber via SPP excitation to the outer air-metal interface of gold conical tip with high efficiency and independent on the incident light polarization. Such a 3H SNOM tip and SNOM instrument would open up a new frontier of high spatial-temporal resolution detecting, imaging, and monitoring of single-molecule physical, chemical, and biological systems, and deepen our understanding of their basic science in the single-molecule level.

## Materials and methods

### Sample fabrications

The direct-laser writing (DLW) 3D printing nanofabrication technology used in this study is based on two-photon polymerization (2PP) technique that is implemented by using a commercial instrument system (Photonic Professional, Nanoscribe GmbH), and by a homemade magnetron-sputtering technique. In fabrication, a 780 nm femtosecond laser beam (with pulse width 120 fs and repetition rate 80 MHz) is focused into a negative photoresist (IP-L-780, Nanoscribe GmbH) by a high numerical aperture (NA) oil-immersion objective (63×, NA = 1.4, Zeiss). Detailed operation steps can refer to supplementary information Sec. S1.

### Numerical simulation

#### FDTD simulations of electromagnetic field for 1D grating

To calculate the electromagnetic field distribution for 1D model gold grating, we adopt 2D FDTD technique. Periodic boundary are added along all-directions, the mesh size is 2 nm, and the plane wave of p-polarization is incident at different angles with the wavelength 785 nm. The grating period is 750 nm and the semi-ellipse corrugation width is 580 nm. The gold film has a thickness 80 nm, and the refractive index of the polymer (photoresist) is 1.52.

#### FDTD simulations of electromagnetic field for 3D spiral-grating conical tip

The 3D FDTD method is used for the calculation of electromagnetic fields for the 3D spiral-grating conical tip. Perfectly matched layers (PML) are added along all-directions. The grid size is set as 5 nm to reduce the computation cost. The thickness of gold thin film is 80 nm and the refractive index of the polymer (photoresist) is 1.52. The conical tip is excited by a Gaussian laser beam of a field intensity of 1 Vm^−1^, 10 μm in diameter size and 5 μm in full width at half maximum (FWHM) diameter, in agreement with experiments. Both femtosecond pulse and continuous wave laser are used for full examination of the optical signal transport, SPP excitation and couplings, SPP transport and focus at the tip apex to form a nanofocusing spot. And See more details on methods and materials from supplementary information Sec. S5.

### Supplementary information

Supplementary Information for 3D printing of plasmonic nanofocusing tip enabling high resolution, high throughput and high contrast optical near-field imaging

## Data Availability

All data needed to evaluate the conclusions in the paper are present in the main text.
