# Supplementary material for: 3D printing of plasmonic nanofocusing tip enabling high resolution, high throughput and high contrast optical near-field imaging
Source: Light Sci Appl. 2023 Sep 6;12:219. doi: 10.1038/s41377-023-01272-6 (PMC10483034; doi:10.1038/s41377-023-01272-6)
Supplement: Supplementary file 1 — Supplementary Information for 3D printing of plasmonic nanofocusing tip enabling high resolution, high throughput and high contrast optical near-field imaging [file 41377_2023_1272_MOESM1_ESM.docx]

**Supplementary Information for**

**3D printing of plasmonic nanofocusing tip enabling high resolution, high throughput and high contrast optical near-field imaging**

Li Long^1^, Qiurong Deng^1^, Rongtao Huang^1^, Jiafang Li^2^, and Zhi-Yuan Li^1,3,*^

^1^ School of Physics and Optoelectronics, South China University of Technology, Guangzhou 510641, China

^2^ School of Physics, Beijing Institute of Technology, Beijing 100081, China

^3^ State Key Laboratory of Luminescent Materials and Devices, South China University of Technology, Guangzhou 510640, China

E-mail address: [phzyli@scut.edu.cn](mailto:phzyli@scut.edu.cn)

**Abstract**

**Scanning near-field optical microscopy (SNOM) offers a means to reach a fine spatial resolution down to ~10 nm, but unfortunately suffers from low transmission efficiency of optical signal. Here we present design and 3D printing of a fiber-bound polymer-core/gold-shell spiral-grating conical tip that allows for coupling the inner incident optical signal to the outer surface plasmon polariton with high efficiency, which then adiabatically transport, squeeze, and interfere constructively at the tip apex to form a plasmonic superfocusing spot with tiny size and high brightness. Numerical simulations and optical measurements show that this specially designed and fabricated tip has 10% transmission efficiency, ~5 nm spatial resolution, 20 dB signal-to-noise ratio, and 7000 pixels per second fast scanning speed. This high-resolution, high throughput, and high contrast SNOM would open up a new frontier of high spatial-temporal resolution detecting, imaging, and monitoring of single-molecule physical, chemical, and biological systems, and deepen our understanding of their basic science in the single-molecule level.**

**Keywords: Scanning near-field optical microscopy, 3D printing, surface plasmon polaritons (SPPs)**

**Contents**

**This PDF file includes:**

**Methods:**

**Sec. S1. Sample fabrications**

**Sec.S2. RCWA simulations**

**Sec.S3. Design of Spiral-Grating SNOM Tip**

**Sec.S4. FDTD simulations of electromagnetic field for 1D grating**

**Sec.S5. FDTD simulations of electromagnetic field for 3D spiral-grating conical tip**

**Sec.S6. Explain for detail of SNOM Tip Throughput and Contrast**

**Sec.S7. Numerical simulation for SNOM imaging against a standard sample**

**Sec.S8. Operation of SNOM in illumination mode deng**

**Figures:**

**Fig.S1.** **Schematic geometric diagram of the spiral-grating conical**

**Fig.S2. Numerical simulation characterization of the designed spiral-grating conical tip at optimized wavelength 785 nm**.

**Fig.S3. Numerical simulation characterization of the designed spiral-grating conical tip at under-optimized wavelength 770 nm**.

**Fig.S4. Numerical simulation characterization of the designed spiral-grating conical tip at under-optimized wavelength 800 nm**.

**Fig.S5. Theoretical calculation for SNOM imaging resolution in relation with the nanofocusing spot size.**

**Fig.S6. Diagram of the experimental setup for SNOM imaging.**

**References**

**Methods**

**S1. Sample fabrications**

The direct-laser writing (DLW) 3D printing nanofabrication technology used in this study is based on two-photon polymerization (2PP) technique^1, 2^ that is implemented by using a commercial instrument system (Photonic Professional, Nanoscribe GmbH), and by a homemade magnetron-sputtering technique. In fabrication, a 780 nm femtosecond laser beam (with pulse width 120 fs and repetition rate 80 MHz) is focused into a negative photoresist (IP-L-780, Nanoscribe GmbH) by a high numerical aperture (NA) oil-immersion objective (63×, NA = 1.4, Zeiss). Detailed operation steps can refer to our previous work presented in Ref. 3.^3^ The geometric configuration for the 3D spiral-grating conical gold tip is illustrated in Fig.S1. The overall geometric configuration of the whole spiral-grating conical tip can be looked upon as a semi-ellipse line helically winding around a smooth conical tip in both the polymer template and conformally coated gold film. The appropriate geometric parameters for an optimized spiral-grating conical tip are set as follows: The half conical angle is $\alpha$=20°, the height is *h*=14 $\text{μ}\text{m}$, the pitch of spiral grating is $\Lambda$=750 nm, the grating corrugation shape is a semi-ellipse with horizontal width as 580 nm and vertical height as 300 nm (thus the grating depth is *d=*300 nm), and the thickness of gold thin film is *t=*80 nm. The whole conical tip structure has a bottom diameter of 10.2 $\text{μ}\text{m}$ and a height of 14 $\text{μ}\text{m}$. For better mechanical stability, the conical tip is set to stand tightly at a square plateau of edge length 20 $\text{μ}\text{m}$ and height 5 $\text{μ}\text{m}$.


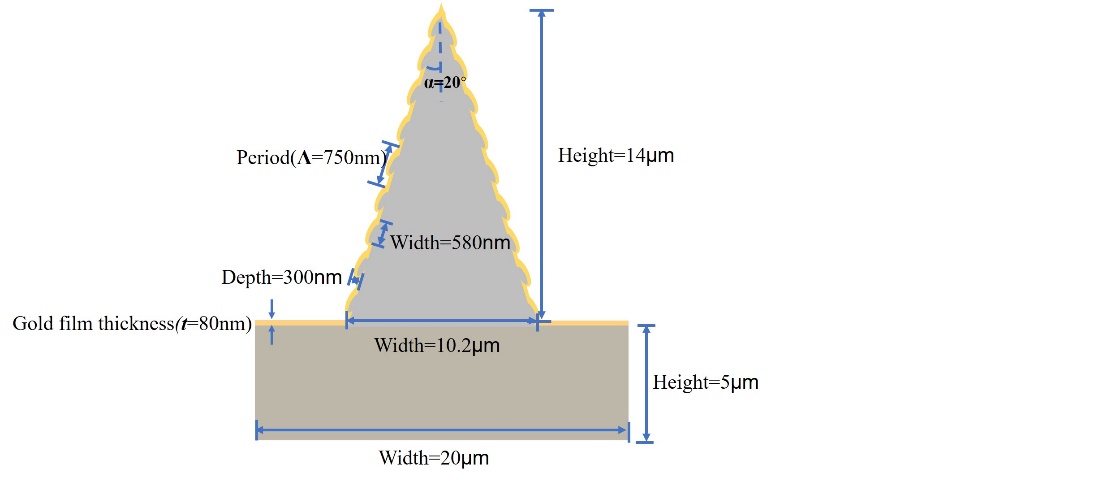


**Fig.S1.** Schematic geometric diagram of the spiral-grating conical solid-core SNOM tip made from a polymer solid core and outer conformally coating gold thin film.

**S2. RCWA simulations**

Rigorous electromagnetic simulations of 1D model gratings discussed in the main text are performed by using the software package GSolver (see http://www.gsolver.com/), which is based on rigorous coupled-wave analysis (RCWA) method.^4^

**S3. Design of Spiral-Grating SNOM Tip**

We are confronted with a six-dimensional parameter space. Surely we have a huge choice of freedom, but also a huge burden to find desirable parameters via 3D finite-difference time-domain (FDTD) rigorous electromagnetic numerical simulation and optimization for such a volumetric 3D SNOM tip involving both dielectric and metal materials. Obviously, a much more efficient alternative way must be explored and adopted for tip design.

For this goal, we explore the major physics processes that make important contribution to the formation of super-bright superfocusing plasmonic hot spot: excitation, tunneling, transporting, squeezing, interfering, and focusing of SPPs, and their good collective action to transform the optical signal from optical fiber mode to the 3H plasmonic focus spot at the tip apex. We find that among these factors, the tunneling of inner optical signal and coupling of outer air-metal-interface SPPs is the crucial one. The special structure of the gold-coated spiral-grating conical tip is adopted mainly to convert the arbitrary polarization optical signals propagating “inside” the fiber core and tip solid core into SPPs on the “outer” gold thin film, which then transport, interfere, and hyperfocus at the sharp apex (Fig. 2A). Previous experiments have demonstrated that the polarization of the internal excitation light has no influence on the intensity of the focused hot spot.^5^ Essentially, it relies on adiabatically converting the dielectric mode to the plasmonic mode via the whole inner surface of metal grating. This coupling mechanism can also be explained as a planar Kretschmann configuration, where phase matching is achieved to excite the air-metal SPPs via the periodic corrugation of gold spiral-grating.

Considering this dominant process of grating-assisted SPPs excitation, we simplify the complex 3D helix tip structure into a 1D grating SPPs coupling device model, as illustrated in Fig. 2B. In fact, one only visualizes a 1D grating when looking at the spiral-grating tip along any conical base line passing through the apex. This 1D structure consists of three parts: one is the inner medium with high refractive index (here is the polymer photoresist: $\varepsilon_{p}=2.31$), the other is the middle metal film of periodic corrugation and dielectric constant $\varepsilon_{m}$, and the third is the outer background medium (air, $\varepsilon_{d}=1$). A beam of light in TM polarization mode is incident from the photoresist and impinges at the inner interface between the photoresist and metal film. Then one part is reflected back into the photoresist, and the other part penetrates into and transmits through the metal film. The light intensity of the transmitted part will decay rapidly in the metal film due to absorption attenuation. Yet, at appropriate conditions of incident angle $\theta_{\text{SPP}}$ and light wavelength $\lambda_{\text{SPP}}$, and if the metal film is not too thick, it is possible to cause resonant tunneling of the incident optical energy and maximum excitation of SPPs propagating along the outer air-metal interface. At these conditions, the reflectance of the incident beam is very low, because almost all the incident light energy is coupled into SPPs propagating along the air-metal interface, if the unavoidable metal absorption loss is neglected.^6-10^ It is well-known that for usual prism SPP coupling device with planar metal film coating, the propagation wave-vector constant of SPPs is given by

$k_{\text{SPP}}=k_{0}\left( \frac{\varepsilon_{d}\varepsilon_{m}}{\varepsilon_{d}+\varepsilon_{m}} \right)^{1/2}=k_{\text{inc}}=\frac{\omega}{c}\sqrt{\varepsilon_{p}}\sin(\theta_{\text{SPP}})$ (1)

Here $k_{0}=2\pi/\lambda_{\text{SPP}}$, $\omega$ is the angular frequency of incident light, and *c* is the light speed in vacuum. For a more flexible metal grating coupler, the momentum matching between SPPs and incident light is approximately given by

$k_{\text{SPP}}=k_{0}\left( \frac{\varepsilon_{d}\varepsilon_{m}}{\varepsilon_{d}+\varepsilon_{m}} \right)^{1/2}=k_{\text{inc}}+qK=\frac{\omega}{c}\sqrt{\varepsilon_{p}}\sin(\theta_{\text{SPP}})+qK,\text{ }q=0,\pm1,\pm2,...$ (2)

Here *q* is the diffraction order, and $K=2\pi/\Lambda$ is the grating vector. This momentum matching formula Eq. (*2*) offers a great flexibility to engineer the geometry of metal grating for efficient excitation of air-metal SPPs.

For convenience of operation for 3D printing technique, we determine the period as *Λ*=750 nm and other geometric parameters that are presented in Methods, Fig. S1. The well-established plasmonic physics model states that the excitation of SPPs rightly corresponds to the minimum reflectance, zero transmittance, and maximum absorbance. Thus, to accurately determine the condition of SPPs excitation beyond the approximate model Eq. (*2*), we scan the wavelength and angle of the incident light and calculate the reflection, transmission, and absorption of incident light against the metal grating by adopting the rigorous coupled-wave theory.^4^ For more details of calculation, see Methods, Sec. S2. The calculation results are shown in Fig. 2C, where the color-bar represents the absorption efficiency and the reddest region represents the largest absorbance and lowest reflectance. The reflectance is the lowest when the incident light wavelength is 785 nm and the incident angle is 70*°*. Thus we judge $\theta_{\text{SPP}}=70^{o}$ and $\lambda_{\text{SPP}}=785 \mathrm{nm}$, and at this condition, more energy is coupled to the outer surface of the metal grating to form SPP evanescent waves. Figure 2D is a 1D plot of transmission, reflection, and absorption spectrum versus the incident angle $\theta$ at the fixed incident wavelength of 785 nm. It can be seen that there appear local dips at points A, B, and C. Notably, point C has the globally minimum reflectance, nearly zero transmittance, and maximum absorbance as high as 86%, which means a large amount of incident energy is coupled to excite the outer air-metal SPPs and eventually dissipates into heat when the SPPs transport.

In order to verify the correctness of the rigorous coupled-wave theory calculation, we use the FDTD method to calculate the 1D electromagnetic field for this 1D metal grating when the incident 785 nm light at angles corresponding to the three points A, B, and C in Fig. 2D are coupled to the outer surface of the metal grating, respectively. For more details of calculation, see Methods, Sec. S3. The results are displayed in Fig. 2E-G, respectively. The mesh size is 2 nm, and periodic boundaries are used in the simulations. The results show that when the incident light wavelength is 785 nm and the incident angle is 70°, much more incident energy is coupled to the outer metal grating surface to form SPP evanescent waves, and indeed at this condition the SPP is excited with the maximum efficiency.

After the 1D grating model parameters are determined, we bring the structural parameters into the realistic 3D spiral-grating design. As shown in Fig. 2A, the light incident angle $\theta_{\text{SPP}}$ is related to the half conical angle $\alpha$ by $\alpha=\pi/2-\theta_{\text{SPP}}$. Thus, the 3D tip parameters are set as: the half conical taper angle is 20°, the gold film thickness is 80 nm, the period is 750 nm, and the incident light wavelength is 785 nm. To confirm the performance of this specific 3D conical tip design in terms of resolution, throughput, and contrast, we perform a systematic numerical study by using the 3D-FDTD rigorous approach. Some crucial calculation results are presented and discussed in Methods, Sec. S4 and Fig. S2-4.

**S4. FDTD simulations of electromagnetic field for 1D grating**

To calculate the electromagnetic field distribution for 1D model gold grating as illustrated in Fig. 2E-G in the main text, we adopt 2D FDTD technique. Periodic boundary are added along all-directions, the mesh size is 2 nm, and the plane wave of p-polarization is incident at different angles with the wavelength 785 nm. The grating period is 750 nm and the semi-ellipse corrugation width is 580 nm. The gold film has a thickness 80 nm, the dielectric constant data of gold are taken from Ref. 11,^11^ and the refractive index of the polymer (photoresist) is 1.52.

**S5. FDTD simulations of electromagnetic field for 3D spiral-grating conical tip**

The 3D FDTD method is used for the calculation of electromagnetic fields for the 3D spiral-grating conical tip whose geometric configuration has been illustrated in Fig. S1 and whose geometric parameters have been presented in Sec. S1. Perfectly matched layers (PML) are added along all-directions. The grid size is set as 5 nm to reduce the computation cost. The thickness of gold thin film is 80 nm. The dielectric constant data of gold are taken from Ref. 3 and the refractive index of the polymer (photoresist) is 1.52. The conical tip is excited by a Gaussian laser beam of a field intensity of 1, 10 $\text{μ}\text{m}$ in diameter size and 5 $\text{μ}\text{m}\text{ }$ in full width at half maximum (FWHM) diameter, in agreement with experiments. Both femtosecond pulse and continuous wave laser are used for full examination of the optical signal transport, SPP excitation and couplings, SPP transport and focus at the tip apex to form a nanofocusing spot. We present a comparison of 3D electromagnetic fields at three wavelengths, namely 785 nm (Fig. S2), 770 nm (Fig. S3), and 800 nm (Fig. S4).

The wavelength of 785 nm rightly matches the design and optimization of SNOM tip and also matches the incident laser wavelength in experiment. The electric field intensity pattern within the 2D XZ cross-sectional plane is displayed in Fig. S2A for the entire SNOM tip. When the incident Gaussian beam impinges upon SNOM tip from inside the polymer core, the two edges of the wavefront first confront the inner side of the gold spiral grating, with one part of energy reflected with an incline angle $\theta_{\text{SPP}}$ (the SPP excitation angle) to the forward direction and another part of energy penetrates through the metal film and collectively excite the outer air-metal SPPs due to good momentum matching. The central part of the laser beam wavefront continues to go forward and interferes with the incline-reflection signal wavefront to form oscillating ripple patterns. The interference between the incline-reflection light from the two edges in this YZ cross section (and concentric edges for the entire conical tip) and the incident light is always constructive right at the central axis of conical tip, leading to a much stronger field intensity here compared with in other regions. More and more incline-reflection forward-propagating light energy and the incident light energy pour forward into the increasingly narrower channel towards the tip apex and make the field intensity become increasingly stronger. At the same time, more and more energy tunnels through the metal film and excite the outer air-metal SPPs. They transport forwards towards the tip apex from the outside surface, interfere constructively with each other from various conical channels in the whole cone surface, make the surface field intensity stronger and stronger, and finally form a plasmonic nanofocusing spot of high brightness in the vicinity of tip apex.

In some sense, the inner space of this conical tip behaves like a funnel for the incident light to adiabatically flow and squeeze into a very strong intensity at its end of inner-channel trip, while the outer surface of this conical tip also behaves like another funnel for the globally excited and coupled SPPs to adiabatically flow and squeeze into a very strong intensity at its end of outer-surface trip and form an ultra-intense plasmonic nanofocusing spot. This is exactly what we find in Fig. S2A. Of course, the outer-channel of SPP excitation, coupling, squeezing, and nanofocusing becomes effective only when the incident light accurately satisfies the momentum matching condition in both wavelength and angle. Close to the tip apex, this momentum matching condition cannot be satisfied perfectly due to the tiny subwavelength space inducing wave vector dispersion and expansion from a single value of *k_z_* for the incident laser beam into a broad band of (*k_x_*, *k_y_*, *k_z_*), thus, the SPP excitation and coupling condition is no longer perfect. Consequently, there appear apparent light signals scattering into a wide range of directions and orientations, and obviously they form the background noise signal for near-field imaging by such a SNOM tip.

Yet, as clearly illustrated in Fig. S2B for the field pattern around the tip apex, such a background noise signal intensity is far weaker than the plasmonic nanofocusing spot. The SPP excitation, propagation, interference, squeezing, and nanofocusing is very apparent in this zoom-in 2 $\text{μ}\text{m}$ 1.5 $\text{μ}\text{m}$ small region, and the very tiny and bright nanofocusing spot ~10 nm away from the tip apex has a very large intensity. To have a clarified idea about the strength of this nanofocusing spot in reference to the background noise signal, we display the field intensity pattern in the XY horizontal cross sectional plane passing through the center of nanofocusing spot in Fig. S2C-E, with a smaller and smaller region (and a larger and larger zoom-in) to reveal more and more details of the nanofocusing spot picture. The center of nanofocusing spot exhibits a huge field intensity as ~29,000, meaning a 4 orders of magnitude huge enhancement factor in this plasmonic hot spot against the incident light intensity (set as 1). The *x*-axis 1D line plot Fig. S2F shows that the nanofocusing spot has a FWHM size as small as 6 nm. Further calculation across a 10 $\text{μ}\text{m}$ output plane (of the same size as the incident laser beam) shows that the total transmitted energy power is 20% of the incident energy power, while about 10% is located within the ~10 nm nanofocusing spot. Indeed, this deliberately designed and optimized spiral-grating conical tip have a very wonderful performance in forming a bright and tiny nanoscale focusing spot and has the potential to serve as an excellent SNOM tip for near-field imaging with high resolution (~6 nm), high throughput (~20%), and high contrast with large signal-to-noise ratio (SNR) (~1000).

Further numerical simulations made at other wavelengths of incident light indicate that the SNOM tip has a pretty good operation bandwidth of about 50 nm in terms of near-field imaging resolution, throughput, and contrast. Two typical examples at 770 nm and 800 nm wavelength are displayed in Fig. S3 and Fig. S4, respectively. The overall field patterns are similar to the case for 785 nm, which means that the outer-channel of SPP excitation, coupling, squeezing, and nanofocusing also are still effective for wavelength some distance away from the optimum value of 785 nm. A bright and tiny plasmonic nanofocusing spot is clearly visualized, with the maximum field intensity reaching 12,000 and 2,100, and the FWHM size reaching 10 nm and 10 nm, at the 770 nm and 800 nm incident wavelength, respectively. As a special notice, 785 nm is just the central wavelength of popular Ti: Sapphire femtosecond pulse laser. A 50 nm operation bandwidth indicates that this designed SNOM tip is suitable for optical near-field imaging and monitoring with both high spatial and temporal resolution when combined with Ti: Sapphire femtosecond pulse laser technology.


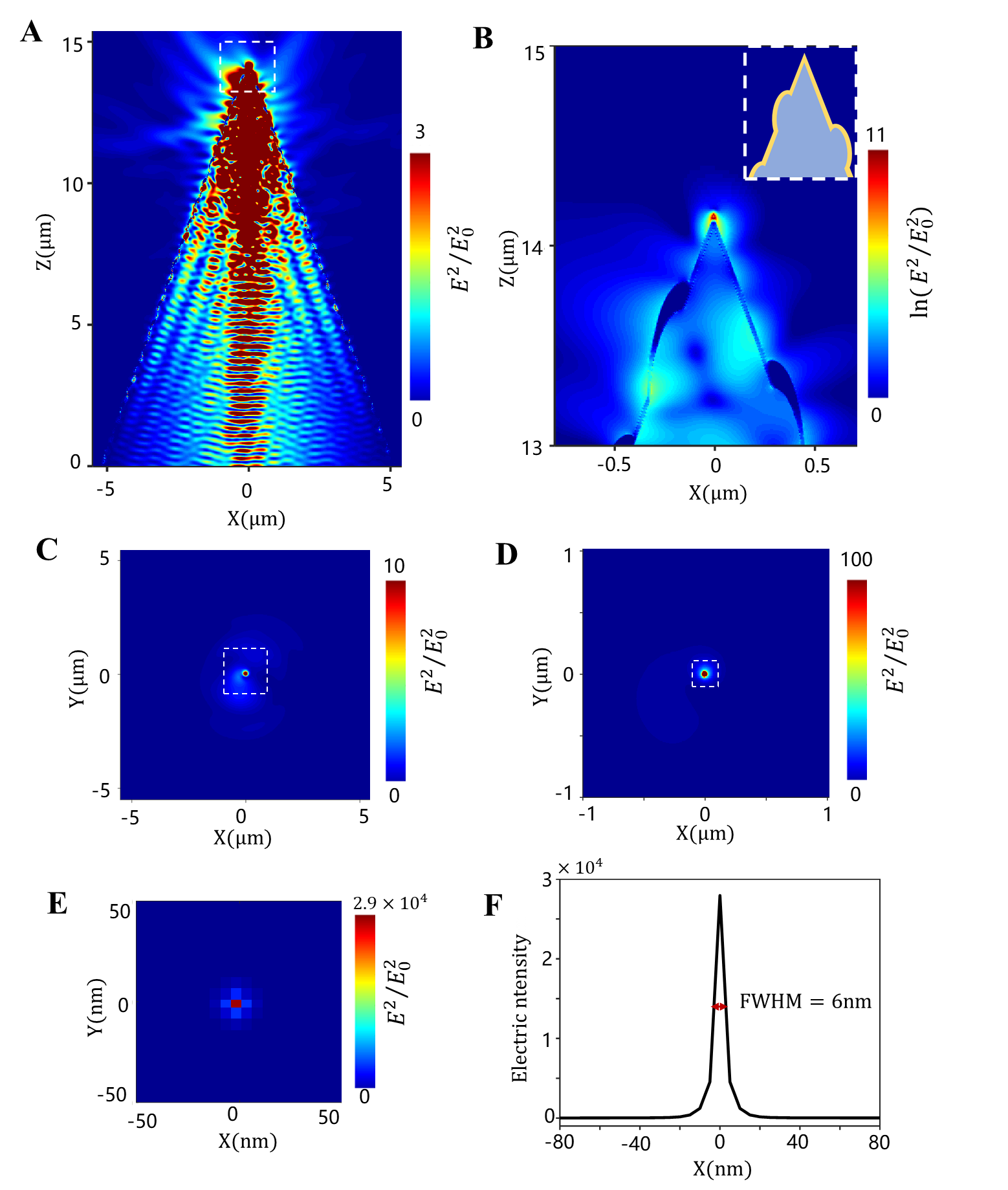


**Fig.S2.** **Numerical simulation characterization of the designed spiral-grating conical tip at optimized wavelength 785 nm**. **(A)** Simulated electric field intensity of the tip in the vertical XZ cross sectional plane; **(B)** The partially enlarged view of the logarithmic-scale field strength distribution within the white dashed square in panel (A). The inset is the corresponding [structure diagram](javascript:;) where the yellow layer and the blue parts represents the gold layer and [photoresist](javascript:;), respectively. **(C)** Simulated electric field intensity in the horizontal XY cross sectional plane 10 nm above the tip vertex and intersecting the nanofocusing spot center. **(D)**, **(E)** The zoomed-in view of the white dashed square in panel (C) and (D), respectively. **(F)** 1D plot of electric field intensity distribution versus the X-coordinate at Y=0 in panel (E)**.**


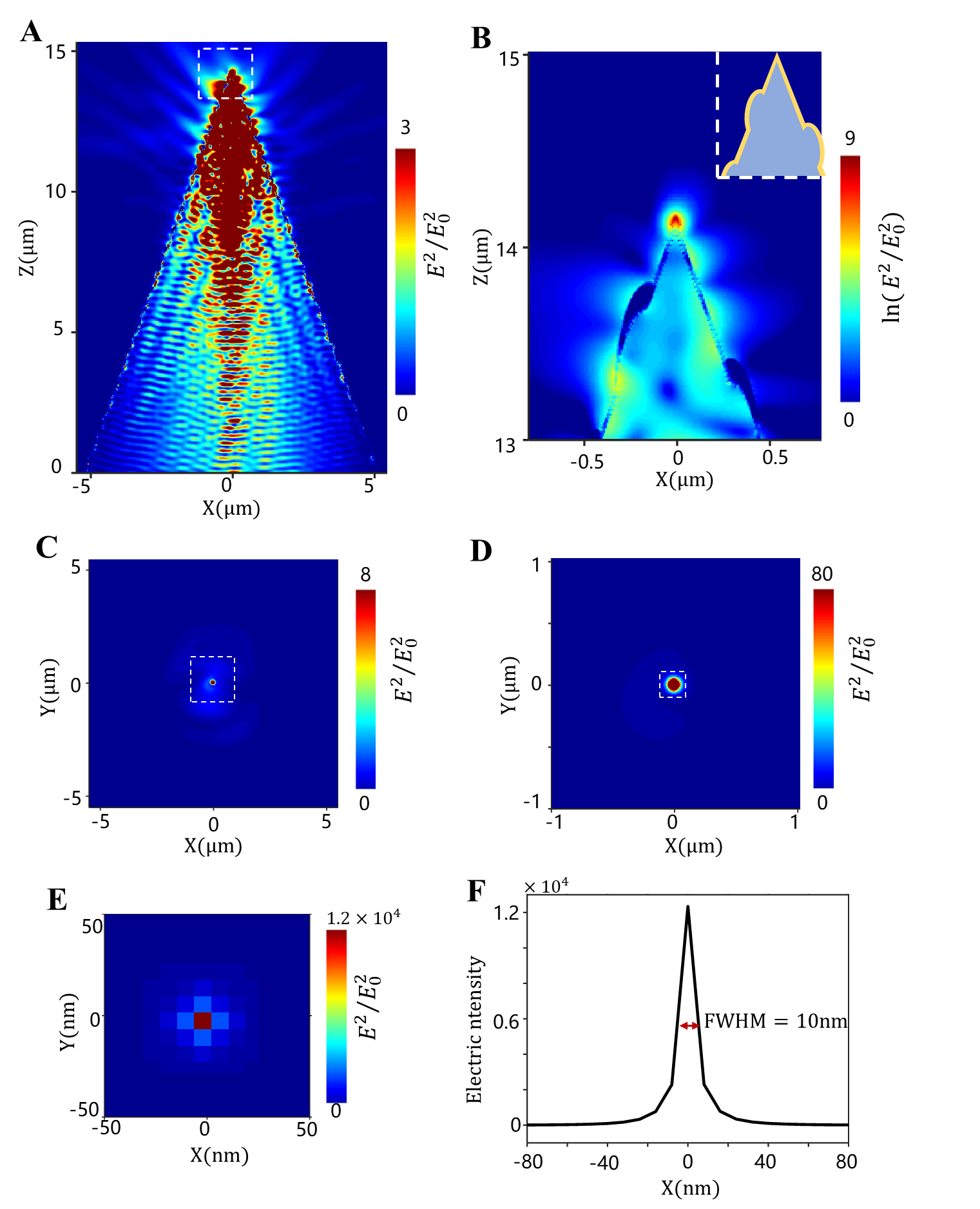


**Fig.S3.** **Numerical simulation characterization of the designed spiral-grating conical tip at under-optimized wavelength 770 nm**. **(A)** Simulated electric field intensity of the tip in the vertical XZ cross sectional plane. **(B)** The partially enlarged view of the logarithmic-scale field strength distribution within the white dashed square in panel (A). The inset is the corresponding [structure diagram](javascript:;) where the yellow layer and the blue parts represents the gold layer and [photoresist](javascript:;), respectively. **(C)** Simulated electric field intensity in the horizontal XY cross sectional plane 10 nm above the tip vertex and intersecting the nanofocusing spot center. **(D)**, **(E)** The zoomed-in view of the white dashed square in panel (C) and (D), respectively. **(F)** 1D plot of electric field intensity distribution versus the X-coordinate at Y=0 in panel (E)**.**


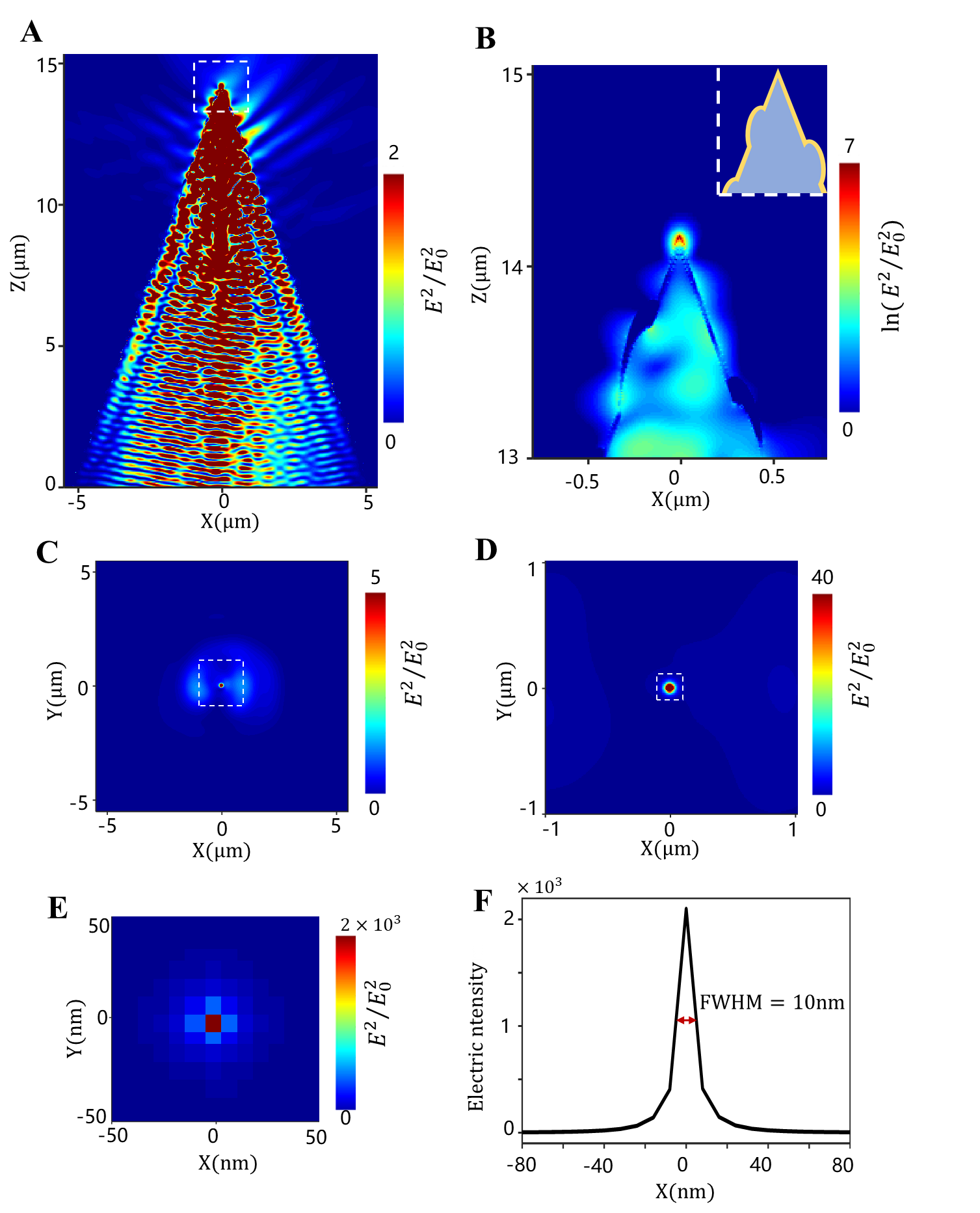


**Fig.S4.** **Numerical simulation characterization of the designed spiral-grating conical tip at under-optimized wavelength 800 nm**. **(A)** Simulated electric field intensity of the tip in the vertical XZ cross sectional plane; **(B)** The partially enlarged view of the logarithmic-scale field strength distribution within the white dashed square in panel (A). The inset is the corresponding [structure diagram](javascript:;) where the yellow layer and the blue parts represents the gold layer and [photoresist](javascript:;), respectively. **(C)** Simulated electric field intensity in the horizontal XY cross sectional plane 10 nm above the tip vertex and intersecting the nanofocusing spot center. **(D)**, **(E)** The zoomed-in view of the white dashed square in panel (C) and (D), respectively. **(F)** 1D plot of electric field intensity distribution versus the X-coordinate at Y=0 in panel (E)**.**

**S6.** **Explain for detail of SNOM Tip Throughput and Contrast**

We first examine the crucial factor of tip throughput by sending 785 nm optical signal to a bare optical fiber with flat-cleaved facet and to the optical fiber binding solid-core spiral-grating conical tip. The optical mode picture for the three samples are displayed in Fig. 3 for both 2D contour plot and 1D scanning plot. The pictures show that the flat-cleaved optical fiber output mode has a regular circular profile, with a modal diameter of about 10 $\text{μ}\text{m}$ and FWHM diameter about 5 $\text{μ}\text{m}$, while the solid-core spiral-grating tip has a regular model profile but has a much smaller size as FWHM ~1 $\text{μ}\text{m}$. In comparison, the hollow-core spiral-grating tip shows a complicated concentric-ripple modal pattern and much larger size than the solid-core tip. It can be seen that the energy power for the solid-core tip is highly concentrated at the center of tip and the residual energy power in the background is very small. This means the solid-core tip has a much smaller focusing spot size and much higher signal contrast and SNR compared with the optical fiber and hollow-core tip when used as a SNOM tip for optical near-field imaging. SNR is measure of signal contrast quality, it is related to signal power ($P_{signal}$) and noise power ($P_{noise}$) by formula $\text{SNR=10lo}\text{g}_{10}(\frac{P_{signal}}{P_{nosie}})$ represented in the unit of decibel (dB). In Fig. 3D-E, it can be seen that the solid-core spiral-grating conical tip has an SNR of 1/0.01=100=20 dB in the X direction and an SNR of 1/0.004=250=24 dB in the Y direction. In fact, the experimental SNR is already quite high, but still one order of magnitude smaller than the theoretical calculation value of SNR=30~40 dB as shown in Fig. S2E.

**S7. Numerical simulation for SNOM imaging against a standard sample**

We consider what is observed when we use a nanofocusing light spot as an illumination source for optical near-field imaging by using SNOM against a specific sample involving with nanoscale structural features. The structure of a model sample is schematically illustrated in Fig. S5A. We take the 60 nm chromium film engraved with an 80 nm wide rectangular groove as the standard sample, because chromium is a bad metal that is strongly absorptive and does not exhibit considerable plasmon resonance. In some sense, chromium is a black metal. The groove sample is illuminated by a nanofocusing spot with different spot sizes as light sources. The point-like light source (here line-like 1D source) is 10 nm above the sample surface and is scanned along the X-axis direction, namely perpendicular to the groove. A planar monitor is placed right below the grating sample to collect all the transmitted light signal and record the energy power value for each point-source scanning position, similar to the operation mode considered in this work, namely, an ordinary SNOM working in the illumination mode. The calculated 1D curve of the scanning results is shown in Fig. S5B. The results show that the near-field imaging resolution is related to the spot size. As the spot size decreases, the power to reveal the ideally steep edge of the rectangular chromium groove is increased, which is rightly a clear signature that the imaging resolution is also increasing. When the spot size is 8 nm, the imaging resolution is also 8 nm. It can thus be fairly well concluded that the most efficient way to improve the spatial resolution of SNOM near-field imaging is to reduce the nanofocusing spot size to an as small as possible value.

**
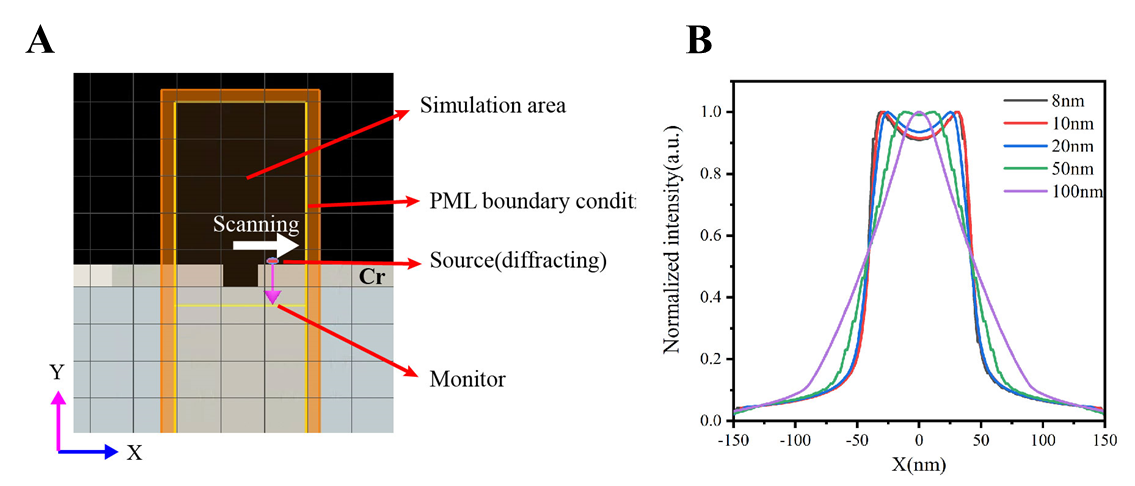
**

**Fig.S5**. **Theoretical calculation for SNOM imaging resolution in relation with the nanofocusing spot size. (A)** Simulation structure model of 1D lamellar grating of grooves in chromium thin film. **(B)** Simulation result of near-field scanning imaging of the 1D chromium grating with different spot sizes as 8 nm, 10 nm, 20 nm, 50 nm, and 100 nm, respectively.

**S8. Operation of SNOM in** **illumination mode**

The schematic configuration of experiment setup for optical near-field imaging via homemade SNOM tip in association of a commercial SNOM machine (NTEGRA Solaris SNOM, NT-MDT) is illustrated in Fig. S6. The laser light at 785 nm coming from the far end of the optical fiber transports to illuminate the inner side of the conical tip and generate a highly localized hot spot to serve as the illumination source for near-field imaging. We adjust the position of the far end of the optical fiber to maximize the coupling efficiency of the incident laser light into the fiber. Then we monitor and align the relative vertical position of sample and SNOM tip by sending the white light to illuminate the sample-tip system and observing them via the camera, which, together with a tuning fork for atomic force measurement, is connected with a computer feedback system to control the vertical position of tip to locate in the near-field region of the sample. We drop the SNOM tip to 10 nm in vertical distance from the sample surface and then scan in the horizontal plane to illuminate the sample with a scanning speed of 15 $\text{μ}\text{m}$ s-1 and a step size of 2 nm. The point-like light emitting from the SNOM tip passes through the sample. The transmission light carries the local optical property of the sample, is collected by a $20\times$ objective lens, and finally goes to the PMT to serve as the recorded near-field optical signal intensity at every scanning position in the 2D XY horizontal plane. The signal intensity as a function of the XY scanning position is just the near-field contour plot as shown in Fig. 4 and Fig. 5 in the main text.


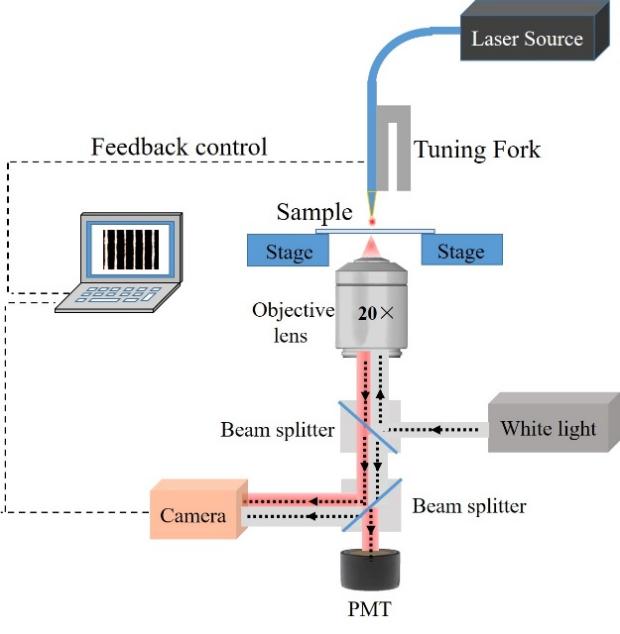


**Fig.S6.** **Diagram of the experimental setup for SNOM imaging.**

**References**

1. Kawata, S.; Sun, H.-B.; Tanaka, T.; Takada, K., Finer features for functional microdevices. *Nature* **2001,** *412* (6848), 697-698.

2. Jia, B.; Li, J.; Gu, M., Two-Photon Polymerization for Three-Dimensional Photonic Devices in Polymers and Nanocomposites. *Australian Journal of Chemistry* **2007,** *60* (7), 484-495.

3. Long, L.; Deng, Q.; Huang, R.; Li, Z.-Y., Plasmonic enhanced fluorescence via 3D printing spiral conical tapered gold tip bound to optical fiber. *APL Photonics* **2022,** *7* (4), 046107.

4. Auslender, M.; Hava, S., Scattering-matrix propagation algorithm in full-vectorial optics of multilayer grating structures. *Opt. Lett.* **1996,** *21* (21), 1765-1767.

5. Li, J.; Mu, J.; Wang, B.; Ding, W.; Liu, J.; Guo, H.; Li, W.; Gu, C.; Li, Z.-Y., Direct laser writing of symmetry-broken spiral tapers for polarization-insensitive three-dimensional plasmonic focusing. *Laser & Photonics Reviews* **2014,** *8* (4), 602-609.

6. Kim, S.; Yu, N.; Ma, X.; Zhu, Y.; Liu, Q.; Liu, M.; Yan, R., High external-efficiency nanofocusing for lens-free near-field optical nanoscopy. *Nature Photonics* **2019,** *13* (9), 636-643.

7. Stockman, M. I., Nanofocusing of Optical Energy in Tapered Plasmonic Waveguides. *Physical Review Letters* **2004,** *93* (13), 137404.

8. Issa, N. A.; Guckenberger, R., Optical Nanofocusing on Tapered Metallic Waveguides. *Plasmonics* **2007,** *2* (1), 31-37.

9. Ding, W.; Andrews, S. R.; Maier, S. A., Internal excitation and superfocusing of surface plasmon polaritons on a silver-coated optical fiber tip. *Physical Review A* **2007,** *75* (6), 063822.

10. Giugni, A.; Torre, B.; Toma, A.; Francardi, M.; Malerba, M.; Alabastri, A.; Proietti Zaccaria, R.; Stockman, M. I.; Di Fabrizio, E., Hot-electron nanoscopy using adiabatic compression of surface plasmons. *Nature Nanotechnology* **2013,** *8* (11), 845-852.

11. Weber, M. J., *Handbook of optical materials*. CRC press: 2002; Vol. 19.
